# Using Machine Vision of Glycolytic Elements to Predict Breast Cancer Recurrences: Design and Implementation

**DOI:** 10.3390/metabo13010041

**Published:** 2022-12-27

**Authors:** Howard R. Petty

**Affiliations:** Department of Ophthalmology and Visual Sciences, University of Michigan Medical School, 1000 Wall Street, Ann Arbor, MI 48105, USA; hpetty@umich.edu

**Keywords:** computer-assisted diagnosis, machine learning, glycolysis, breast cancer recurrences, phospho-Ser226-glucose transporter type 1, phosphofructokinase type L

## Abstract

A major goal of biomedical research has been the early and quantitative identification of patients who will subsequently experience a cancer recurrence. In this review, I discuss the ability of glycolytic enzyme and transporter patterns within tissues to detect sub-populations of cells within ductal carcinoma in situ (DCIS) lesions that specifically precede cancer recurrences. The test uses conventional formalin fixed paraffin embedded tissue samples. The accuracy of this machine vision test rests on the identification of relevant glycolytic components that promote enhanced glycolysis (phospho-Ser226-glucose transporter type 1 (phospho-Ser226-GLUT1) and phosphofructokinase type L (PFKL)), their trafficking in tumor cells and tissues as judged by computer vision, and their high signal-to-noise levels. For each patient, machine vision stratifies micrographs from each lesion as the probability that the lesion originated from a recurrent sample. This stratification method removes overlap between the predicted recurrent and non-recurrent patients, which eliminates distribution-dependent false positives and false negatives. The method identifies computationally negative samples as non-recurrent and computationally positive samples are recurrent; computationally positive non-recurrent samples are likely due to mastectomies. The early phosphorylation and isoform switching events, spatial locations and clustering constitute important steps in metabolic reprogramming. This work also illuminates mechanistic steps occurring prior to a recurrence, which may contribute to the development of new drugs.

## 1. Introduction

As metabolism is a border between life and death, its importance can hardly be overestimated. Changes in metabolism are also important because they enable specific physiological changes. One broadly important metabolic change is the acquisition of aerobic glycolysis, the catabolism of glucose to lactate in the presence of oxygen (1–3). For example, aerobic glycolysis is associated with cell development and adaptive immunity [1,2]. Since Warburg’s seminal 1927 paper [3], we have known that rapid glucose utilization and the release of protons are indicative of aggressive cancer. The role of aerobic glycolysis in cancer is supported by nearly 100 years of research [4]. From a clinical perspective, the role of glucose uptake in cancer is supported by the ability of fluorodeoxyglucose and positron emission tomography to identify human cancers in vivo [5]. It is likely that regulatory changes are required at rate-controlling steps of glycolysis to provide the observed enhancement of glucose uptake. To better understand the mechanism of aggressive (recurrent) cancer and to develop a computer-assisted diagnostic (CAD) test, we have performed retrospective studies using pre-invasive ductal carcinoma in situ (DCIS) samples from women who will or will not experience a cancer recurrence [6,7,8,9,10]. Using immunofluorescence microscopy, these tissue samples were tested with antibodies directed against specifically modified forms of glycolytic elements (phosphorylated proteins and enzyme isoforms [8]). By training computers with images of glycolytic components of interest in tissue sections from recurrent and non-recurrent patients, one can identify important glycolytic elements participating in metabolic preparations for enhanced glycolysis and, simultaneously, develop a computer vision test for recurrent disease to assist in patient management.

## 2. Considerations

To design a clinical test, several factors must be considered. For example, pathology samples are processed and stored as formalin-fixed and paraffin-embedded (FFPE) tissue samples. Consequently, there are no small molecules, such as metabolites, available to study. However, FFPE-treated samples retain most antigens after antigen retrieval and their spatial locations. Therefore, we indirectly assess metabolites by following the regulatory changes in glycolytic enzymes such as phosphorylation, and their physical properties, such as the patterns observed during storage, intracellular transit, and co-clustering of these macromolecules.

The signal/noise ratio and the propagation of error are important considerations for any scientific experiment. If the signal-to-noise ratio is low, then it will be difficult or impossible to distinguish two populations. For example, the stochastic fluctuations of gene expression cause large variations in expression levels [11,12]. The heterogeneity of the intraductal and extraductal spaces are additional sources of uncertainty [13,14]. Of course, sample heterogeneity will dilute the signal of interest. For these reasons, it is advisable to have high signal-to-noise ratios in the raw data, as noise will not be improved by analysis. Using the high signal-to-noise ratio and spatial resolution of fluorescence microscopy and the strengths of machine learning in pattern recognition, issues regarding stochastic fluctuation in biomarker expression and sample heterogeneity are minimized.

**Figure 1:**
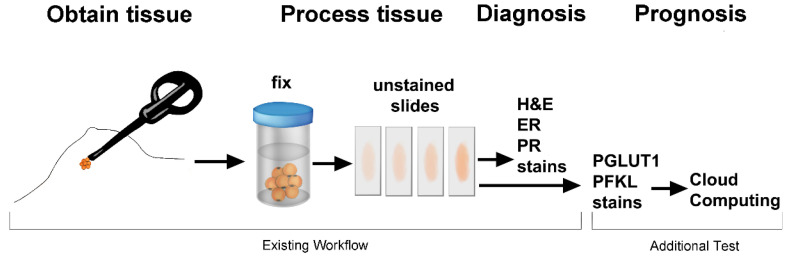
Workflow from patient to cloud computing. Tissue sections are stained with H&E and in some cases for estrogen and progesterone receptors. Implementation of this test only requires two additional stains to assess glycolytic enzyme patterns followed by cloud computation of the patient’s outcome.

Another important consideration in assay design is clinical workflow (Figure 1). If the workflow is difficult or inefficient, the test will not be used. Tissue sections are stained with anti-phospho-Ser226-GLUT1 and anti-PFKL in the laboratory. Images of tissue labeled with anti-phospho-Ser226-GLUT1 and anti-PFKL are uploaded to the cloud to obtain outcome predictions. This approach is favored for many reasons, including the fact that it is easier to maintain and update software at a single portal and that quality control of input data can be performed.

**Figure 2:**
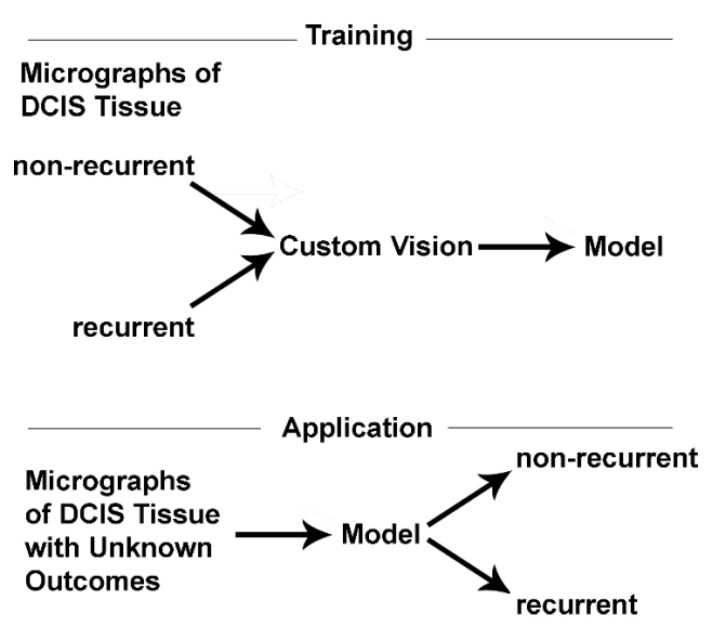
A broad illustration of the steps involved in the training and application phases of machine learning are shown. These studies constitute a binary classifier wherein training is performed using images of known non-recurrent and recurrent samples. The models developed during the training phase are tested in the application phase using holdout data that were not used in the creation of the models.

To understand aggressive cancer, one must understand Warburg’s effect, and to understand that you must first understand the mechanism of heightened glucose flux. Instead of studying the enzymes mediating glucose flux under normal conditions, we focused on unusual forms of glycolytic components linked to unusually rapid glycolytic activity. We found that certain patterns of phospho-Ser226-GLUT1 and PFKL, two unique glycolytic elements that accelerate glycolysis, could predict patient recurrences. To assay biomarker performance in detecting recurrent disease, we employed machine learning. Supervised machine learning was used to create models to predict patient outcomes using Azure’s Custom Vision application (Microsoft, Inc., Redmond, WA, USA), as illustrated in Figure 2. Several models were developed over the course of the studies reviewed herein. Only findings of the two best performing models are discussed below. (For details, see references [6,7,8,9,10]).

## 3. Unique Enzyme Patterns Are Found in Pre-Invasive Lesions Prior to Cancer Recurrences

**Figure 3:**
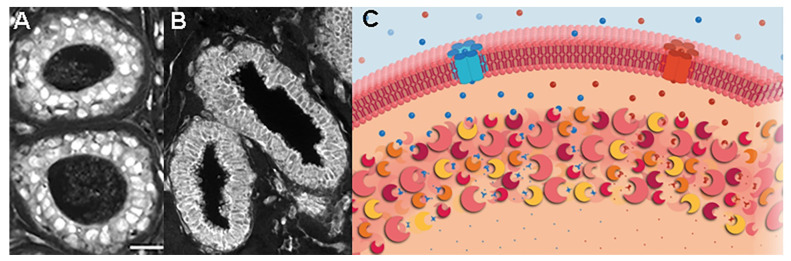
Phospho-Ser226-GLUT1 trafficking in pre-invasive lesions, (**A**) non-recurrent DCIS, (**B**) recurrent DCIS. Phospho-Ser226-GLUT1 and other enzymes accumulate at the cell periphery to form metabolic platforms near tumor cell plasma membranes (**B**,**C**). Due to their physical proximity with one another (i.e., high local concentrations), these enzyme aggregates increase chemical reaction rates of constituent pathways and change cell fates (e.g., a 3-step pathway accelerates flux 110-fold) (Panel C from: Petty, 2022, AJP: Cell Physiol. 322:C991) (Bar = 25 μm, (**A**), lower right).

Although there are several unique features of DCIS lesions prior to recurrences, the most prominent are metabolic platforms. Metabolic platforms are found in regions of the cytoplasm near the tumor cell periphery. In addition to DCIS tissue linked with cancer recurrences, metabolic platforms are predominant structures in breast cancer metastases [6,7]. Ducts of patients who did not experience a recurrence displayed phospho-Ser226-GLUT1 labeling in central region of cells (Figure 3A). Phospho-Ser266-GLUT1 in the nucleus and cytoplasmic vesicles may represent sequestration sites and/or staging areas within epithelial cells. In contrast, Figure 3B shows DCIS tissue stained for phospho-Ser226-GLUT1, which shows a largely peripheral distribution. The delivery of phospho-Ser226-GLUT1 to the cell periphery is important because glucose transport is a rate-limiting step in glycolysis and GLUT1′s V_max_ is accelerated 5-fold by phosphorylation at residue 226 in comparison the non-phosphorylated Ser226 form of GLUT1 [15]. In addition to phospho-Ser226-GLUT1, additional enzymes of glycolysis, the pentose phosphate pathway (PPP), and the glutathione synthesis pathway accumulate at the cell periphery [6]. An immediate consequence of enzyme co-clustering is a dramatically enhanced catalytic throughput: a two-step reaction pathway is accelerated 6-fold whereas a 3-step reaction pathway is accelerated 110-fold [16]. The role of enzyme agglomeration and other steps in cancer recurrence are discussed in Petty [8]. Glycolysis is accelerated by the use of phospho-Ser226-GLUT1 and the PFKL isoform and by their clustering. Glycolysis is also accelerated by PPP and glutathione synthetase accumulation at metabolic platforms because these pathways remove electrons from the cytosol, thus recycling NAD+ for glycolysis. The translocation of phospho-Ser266-GLUT1 and PFKL to the vicinity of the plasma membrane allows tumors the ability to build metabolons at the plasma membrane to rapidly internalize and metabolize glucose. The disposition of multiple pathways at the plasma membrane allows these enzymes to preferentially metabolize molecules immediately upon entering.

**Figure 4:**
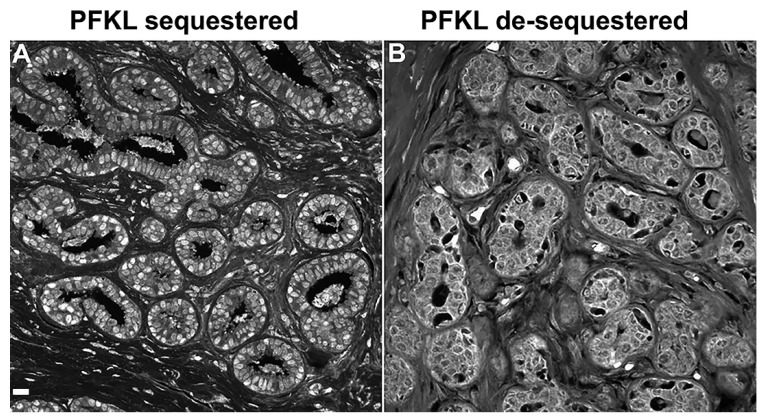
PFKL labeling of tissue sections from patients who would later be determined to be a non-recurrent (**A**) or a recurrent (**B**) patient. Note that PFKL is sequestered in (**A**) and de-sequestered in (**B**). Accumulation at the periphery of cells after de-sequestration in (**B**) suggests that PFKL can participate in metabolon formation with glycolytic elements at the plasma membrane (Bar = 25 μm, lower right).

**Figure 5:**
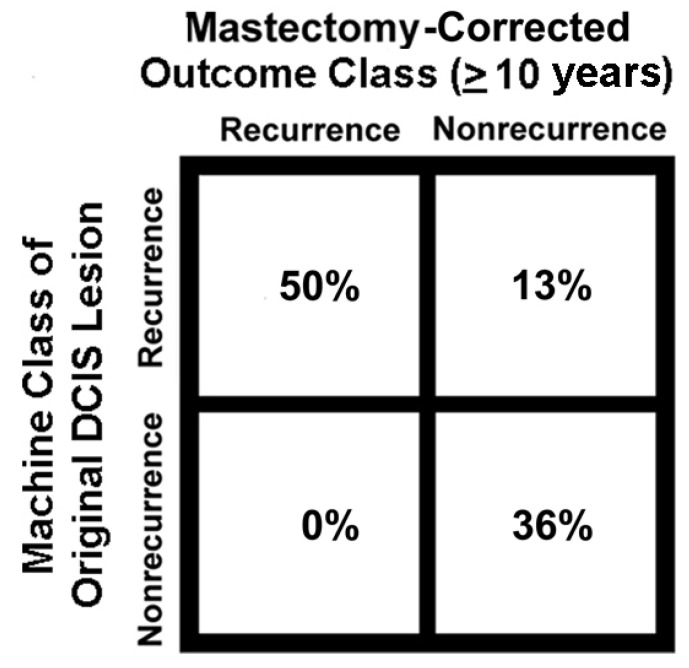
Confusion matrix showing the relationship between machine classification of DCIS cases and patient outcomes. It is important to note that all patients were analyzed by the same computer models for phospho-Ser226-GLUT1 and PFKL.

PFK was studied because it has many advantages as a glycolytic biomarker: complex localization patterns (nuclei, plasma membrane, granules, cytoskeleton, etc.), and it is a rate-controlling step in glycolysis. The PFKL isoform is of particular interest because it lacks elements controlling feedback inhibition of its activity and it is feedback activated by its product fructose (1, 6) bisphosphate. Blocking inhibitor activity and activation by ATP and its product accelerates PFKL, compared to PFKM and PFKP—in addition to the effects of co-clustering. During recurrent DCIS, PFKL, which is normally found in nucleoli near the histone H2A.X [7], is de-sequestered from nucleoli to accumulate near the cell periphery (Figure 4). Thus, catalytic activity is increased by phosphorylation of GLUT1 at Ser-226, accumulation of PFKL at the plasma membrane and by the agglomeration of multiple glycolytic elements at the plasma membrane. Computational analyses of both phospho-Ser226-GLUT1 and PFKL are required to avoid false negatives (Figure 5). If phospho-Ser226-GLUT1 and/or PFKL are computationally positive, the patient is judged to be positive for recurrence.

Phospho-Ser226-GLUT1 and PFKL labeling patterns (Figure 3A,B and Figure 4) of holdout samples were evaluated with computer models (Figure 2) to predict patient outcomes. These outcomes were summed then plotted in the confusion matrix of Figure 5. The confusion matrix used all holdout studies (N = 175) and the same computational models for phospho-Ser226-GLUT1 and PFKL-based outcome predictions; thus, this figure is a recalculation of patient outcomes, not a compilation of patient outcomes reported in separate papers. This includes patient holdout experiments wherein these patients’ micrographs were not used in computer training and micrograph holdout experiments in which specific micrographs used in the holdout experiments were not used for training. As indicated in Figure 5, the results were corrected for mastectomies. This is necessary because many mastectomies are successful, and the patients will therefore appear as computationally recurrent patients who are clinically non-recurrent. The adjustment was determined by subtracting the percentage of DCIS patients who recur after partial mastectomies (10%) [17] from the percentage of patients who recur after treatment with biopsy alone (i.e., without mastectomies) (≅50%) [18,19,20,21]. When this 2 × 2 contingency table was analyzed by Fisher’s exact test, *p* < 0.0001 was obtained. The absence of false negatives indicates that no ill women go undetected. Along this same line of reasoning, it should be noted that all the computed non-recurrent samples were in the clinical non-recurrent group of patients. This group of patients will not have a cancer recurrence, and therefore are overdiagnosed and require no intervention.

Figure 5 shows the presence of a number of false positive samples after an appropriate correction for the effect of mastectomies on patient outcomes. As the test was designed to detect recurrent disease at the earliest possible time, two early steps involving pattern changes of phospho-Ser226-GLUT1 and PFKL were used. The strategy worked because no false negatives were observed. Although rare, cancer recurrences are substantially more complicated that just two proteins. It is possible that a functional defect downstream from these early events blocked recurrences. One speculative possibility is that issues with the monocarboxylate transporters (1 and/or 4) could influence the intracellular pH and redox properties of cancer cells thereby inhibiting cell viability before an overt recurrence took place. Another intriguing possibility is that the epithelial cells in some false positive patients were competent to become invasive breast cancer, but a deficiency elsewhere in the tissue blocked the further development of invasive disease (see Section 4 and Table 1, below). One might imagine that a failure to increase the velocity of glucose efflux from local vessels would diminish an epithelial cell’s ability to initiate the Warburg effect. As there are many ways in which the tissue environment could influence patient outcome, the tumor cell environment could be a rich source of new pharmaceutical targets.

The heterogeneity of tumors has been widely discussed, and the early form of breast cancer DCIS is no exception [22]. In our studies, we can provide quantitative insights on the extent of heterogeneity based on the percentage of recurrence predictions per tissue section. These studies reveal that approximately 4 to 10% of the microscopic fields captured are computed to be positive for recurrences. However, the “aggressiveness” of constituent tissue may be observed to be as high as ~50%. Thus, the in vivo properties of human lesions clearly support the reported heterogeneity of early aggressive cancers.

## 4. Putting Cancer Recurrences into a Biological Perspective

Studies that focus upon tumor cell properties to the exclusion of other tissue components are seriously flawed. Consider the transporter phospho-Ser226-GLUT1. As shown in Figure 3B, phospho-Ser226-GLUT1 may be found at the periphery of ductal epithelial cells in breast lesions of DCIS patients who will subsequently experience a recurrence. However, this is neither a necessary nor sufficient condition for a computed recurrence prediction. Indeed, in some cases the entire duct can be removed from an image of recurrent tissue, and the classifier will still predict a cancer recurrence (7). Similarly, one can clip images of phospho-Ser226-GLUT1-stained tumor associated fibroblasts from micrographs of recurrent tissue, then paste them onto non-recurrent tissue images to alter the predicted outcome. We suggest that cancer recurrences are a property of cancerous tissue, not just cancerous epithelial cells. A tentative list of organelles and tissue elements participating in up-regulation/trafficking of local phospho-Ser226-GLUT1 during recurrences is provided in Table 1. This may seem like a needlessly complicated affair, but it is not. To release more glucose into the interstitial space, the cardiovascular system must up-regulate GLUTs including phospho-Ser226-GLUT1. Interstitial glucose may be used by cancer associated fibroblasts to create metabolites to fuel mitochondrial generation of ATP and other precursor molecules required for tumor growth [23]. Interstitial glucose is also transported across myoepithelial cells and the basal surface of epithelial cells to reach the interior of cancer cells. Epithelial cells rapidly metabolize glucose to keep its intracellular levels close to zero, thus maximizing its chemical potential across tumor cell membranes. What happens if glucose is not available in the extracellular environment, but tumor cells need more glucose? We have found that tumor cells remove metabolons (containing, for example, phospho-Ser226-GLUT1 and PFKL) from their basolateral surface then aggregate the metabolons at the apical surface facing the ductal fluid (milk) [7]. In younger women, milk is always being produced and recycled. Tumors, however, apparently can take advantage of this situation by harvesting glucose, lactose, and other nutrients from the milk and using these nutrients to support tumor growth.

## 5. Barriers to Progress

Significant barriers to progress must be surmounted before CAD software can be broadly applied in the clinic. The single biggest barrier to medical software development is clinical sample sourcing. Most institutions do not make de-identified and annotated metadata of tissue samples available to others. Hospitals and medical systems that work with software developers should be given priority by developers when the app becomes available. A clearinghouse of samples is needed to accelerate research in this field. Additionally, as we have recently reported [10], it is important to check classifier performance with women of various racial backgrounds. Thus, women of different races and ethnicities should be examined in patient holdout studies for proper outcome prediction [10].

Government regulations are another potential impediment to CAD distribution via the cloud. In addition to medical device approvals for the software, the EU’s General Data Protection Regulation (GDPR) is of some concern. The GDPR regulates data transfer, including medical data, between countries. Thus, CAD suppliers operating as software-as-a-service companies may have some difficulty in evaluating samples from EU members.

## 6. Potential Clinical Applications

There are several potential clinical applications for the CAD software described above. One application will be the identification of overdiagnosed patients. According to our recent study of 175 DCIS patients, about 36% of DCIS patients are overdiagnosed; the remainder requires surgery [10]. This application will reduce the unnecessary burdens of surgery including financial costs, disfigurement, pain, sensory disturbances, and psychological damage [24,25]. It is also possible that radiotherapy could cause secondary tumors in normal breast tissue. However, there are also clinical advantages to the identification of patients who will experience a recurrence. We have reported that it is possible to identify normal adjacent tissue as recurrence positive using computer analysis before changes in H&E staining can be observed [7]. This is consistent with proteomic studies that have identified normal adjacent tissue as “tumor-like” [26]. Consequently, computer vision reveals an earlier time point in the life history of a recurrent DCIS lesion than H&E staining. If there is one region with computationally positive normal adjacent tissue (which are likely to be caused by exosomes or lncRNA (8)), there are likely to be many others. It would seem prudent to recommend full mastectomies to patients with computationally positive DCIS lesions and computationally positive normal adjacent tissue(s) (Table 2).

We have performed preliminary studies of ADH, a likely precursor lesion of DCIS that is morphologically similar to DCIS. Using the phospho-Ser226-GLUT1 classifier, we can successfully identify 75% of the ADH patients who will subsequently experience a recurrence. Although this tool has not been optimized for ADH patients, the current form could remove many women from the ADH-DCIS-invasive cancer-metastatic cancer series of events. Thus, patients with positive computational findings for ADH lesions could be treated with lumpectomies. Although computationally positive normal adjacent tissue is unlikely at this stage of tumor development, one could monitor tissues for computationally positive normal adjacent tissue in ADH and, if found, a full mastectomy may be warranted.

The preceding discussion has focused on identifying recurrent and non-recurrent forms of early breast cancer. The mechanism underlying aerobic glycolysis is likely to be similar or identical across tissue types. Hence, it is likely that the software described above, with minor modifications, can be used to assess recurrence probabilities of early-stage cancers from other tissues. Included among these tissues are early stage (in situ or localized) prostate cancer, lung cancer, stomach cancer, thyroid cancer, cervical cancer, and colon cancer. Indeed, roughly 1.5 million people annually could benefit from the technology described above.

## 7. Potential Research Applications

Biomedical research has spent the last 40 years molecularly dissecting tumor cells; perhaps it is time to start putting the cells back together by asking clear physiological and mechanistic questions. In Section 4 of this article, we discuss how tumor cells and their environment participate in determining the outcome of a patient—as judged by machine learning. This opens a new field of in silico cancer biology, wherein we can assess how manipulation of input information effects outcome predictions. The locations of specific proteins during cancer recurrences can be assessed and demonstrated to be relevant to cancer recurrence predictions (Table 1). This information can be gleaned from multiple complementary approaches including composite images, saliency mapping, and deep visualization. (Composite images were briefly discussed in Section 4. In saliency images, the values of each pixel in an image are sequentially changed, and pixels where the change flips the outcome prediction are mapped-thus creating an image of pixels determining patient outcome predictions. In deep visualization information flows in the opposite direction through the classifier, which generates an image of what the computer finds important.) As successful cancer recurrences require the participation of multiple systems within tissues and multiple organelles within cells, the application of these tools will permit the construction of three-dimensional tissue models of rate-controlling steps. Such maps combined with the underlying chemistry will permit next-generation computer simulations that should lead to a far better mechanistic appreciation of cancer recurrences and provide a rational basis for the design of next generation drugs.

A longstanding goal of cancer research has been to develop a molecular understanding of disease. For example, using gene expression data, it is not possible to distinguish between DCIS patients whose lesions will lead to a cancer recurrence from those lesions that will not. There are at least two ways of understanding this observation. First, gene expression is not relevant to the processes involved in causing recurrences in DCIS patients. Simply stated, recurrences of DCIS patients are not due to changes in what is being expressed, but rather where it is being expressed. The focused effort on “omics” approaches to detect recurrent disease is much like efforts in the 1950s and 60s to isolate a “high-energy” chemical intermediate required for oxidative phosphorylation, which was instead found to be due to a “high-energy” spatial gradient of protons. Second, specific genetic changes in DCIS lesions could be important in DCIS recurrences, but experiments have not been executed in such a way that noise was properly managed. Prominent levels of stochastic noise in expression data make it difficult to isolate specific elements. Another source of error is sample heterogeneity. For example, if patient outcomes are assigned after mastectomy, many recurrent patients may appear as non-recurrent patients because they were cured by mastectomy. Given the rate of recurrences after mastectomy and the rate of recurrence in patients not treated by mastectomy (treated with biopsy alone), the probability of comparing a recurrent patient to a non-recurrent patient whose disease was suppressed by mastectomy (a true positive appearing as a non-recurrent patient due to mastectomy) is high; such comparisons are without relevance to disease. Another source of noise is intrasample heterogeneity. For example, in a recurrent DCIS lesion one in 10 or 20 microscope fields predicts recurrence; thus, the signal within the recurrent field is greatly diluted by nearby non-recurrent tissue. Such issues can be avoided by using our machine vision method to identify TP and TN microscope fields. Then, single cell sequencing can be used to obtain only the recurrent fields of a section for subsequent analysis [27]. As multiple tissue factors contribute to a cancer recurrence prediction, extra-ductal mutations could also contribute to the recurrence phenotype; a fundamental topic not yet considered. For example, mutations in CAFs could diminish metabolite export and, thereby, biomass production by tumor cells. Mutations diminishing glucose export from vasculature would also influence tumors. To gauge the nature of intrasample noise, microscopic fields that have been found to be computationally recurrent can be compared to computationally non-recurrent microscope fields in the same section. Inter-sample noise can be assessed by comparing recurrent microscope fields of TPs to lesions of TN patients. Sufficient numbers of such trials should provide new insights. This approach may permit the discovery of pathways responsible for the trafficking and agglomeration of glycolytic elements in cancer.

## 8. Conclusions

Our studies have shown that spatial properties of certain rate-controlling steps of glycolysis, such as a phosphorylated form of glucose transporter 1 and isoform switching of phosphofructokinase are indicative of recurrent cancer. The agglomeration of these components then greatly enhances overall pathway activity by restricting catalysis to a small region of the cell; one can envisage these enzyme clusters as small regions of space (Figure 3C) with extraordinarily high enzyme concentrations and extraordinarily low solvent content. These events will contribute to aerobic glycolysis and can be used to accurately predict breast cancer recurrences.

### 8.1. Machine Vision: An Ideal Diagnostic Tool

In addition to the identification of aggressive pre-invasive cancers, the machine test illustrated above is a fundamental advance in diagnosis/prognosis. As we have previously mentioned [6], the probability of recurrence for most non-recurrent micrographs is between 0–2%. On the other hand, recurrent micrographs may score between 98–100% probability of recurrence. Using a 98% probability of recurrence cutline there is no overlap between the distributions of recurrences and of non-recurrence [10]. The lack of overlap between recurrence probabilities of recurrence and non-recurrence groups is likely due to the classifier software measuring many thousands of data points for each micrograph. These data points include information within and outside the duct. As conventional in vitro tests use one data point such as a colorimetric reading, to infer disease, it cannot compare to machine tests using dozens of micrographs and thousands of data points per micrograph. Consequently, the absence of overlap between these distributions means that there are no FP and FN predicted outcomes due to the software. However, there could be FP and FN due to the samples. Because the patients were treated with mastectomies, patients cured by surgery appear in the data as FP. Thus, using recurrence probabilities to stratify DCIS patients reduces FN and FP. By the judicious choice of biomarkers, many different diagnostic tests could benefit from machine learning methods.

### 8.2. From Computer Bench to Beside

AI/ML-based methods are under study in radiology, cardiology, and ophthalmology. A CAD test to help find prostate cancer has recently been approved by the FDA (Paige.AI, Inc., New York, NY, USA). In this paper we have reviewed a new app that identifies DCIS patients at high risk or low risk for cancer recurrences.

To reach as many patients as quickly as possible, the functionality should be provided via the internet. Thus, distribution via a software-as-a-service business would be advantageous. Both tertiary level care facilities and small rural clinical labs would have access to the same cutting-edge software.

### 8.3. Applications Related to Drug Development

Our studies suggest that the trafficking and co-clustering of specific glycolytic proteins, PPP enzymes, and glutathione synthesis enzymes are key steps in the mechanism of breast cancer recurrences. Thus, agents that block binding at the cell periphery or translocation to the cell periphery may be useful as therapeutics. As illustrated by Table 3 several known anti-cancer agents also display the ability to inhibit intracellular trafficking. For example, taxol promotes PFK dissociation from the cytoskeleton [28,29]. Other reagents may act by disrupting protein trafficking in cells via the cytoskeleton, such as colchicine [30,31,32,33]. For example, colchicine blocks the accumulation of PPP enzymes at the cell periphery [34,35]. Additional trafficking pathways are under study as a treatment. There is little doubt that AI and machine learning will have a major impact on diagnostics and therapeutics.

## Figures and Tables

**Table 1:** Examples of Phospho-Ser226-GLUT1 Image Elements Contributing to Recurrent Outcomes, as Judged by Image Analysis.

Ductal Epithelial Cells	Additional Tissue Sites
Metabolic platforms	Myoepithelial cells
Nucleus/nucleoli	Blood vessels
Cytoplasmic vesicles/enzyme clumps	Tumor-associated fibroblasts

**Table 2:** Possible Clinical Actions for Pre-Invasive Breast Lesions Using Computer Predictions.

Diagnosis	Prognosis		Possible Action
	Computed Recurrence Prediction		
	Lesion	Normal Adjacent	
		Tissue	
**Atypical Ductal** **Hyperplasia**	-	-	none
	+	-	partial mastectomy
**DCIS**	-	-	none
	+	-	partial mastectomy
	+	+	full mastectomy

**Table 3:** Examples of drugs and lead compounds influencing intracellular trafficking [7,8,28,29,30,31,32,33].

Agents	Actions
Taxol	dissociates PFK from cytokeleton
KU55933	blocks GLUT1 translocation
Colchicine	disrupts microtubules, intracellular trafficking
Local anesthetics	disrupts intracellular trafficking; dissociates enzymes from cytoskeleton
Prenylation inhibitor	disrupts membrane association
